# Association between intrafollicular concentration of benzene and outcome of controlled ovarian stimulation in IVF/ICSI cycles: a pilot study

**DOI:** 10.1186/1757-2215-7-67

**Published:** 2014-06-18

**Authors:** Carlo Alviggi, Rossella Guadagni, Alessandro Conforti, Giuseppe Coppola, Silvia Picarelli, Pasquale De Rosa, Roberta Vallone, Ida Strina, Tiziana Pagano, Antonio Mollo, Antonio Acampora, Giuseppe De Placido

**Affiliations:** 1 Dipartimento di Neuroscienze, Scienze Riproduttive ed Odontostomatologiche, Università degli Studi di Napoli ‘Federico II’, Via Sergio Pansini, 5, 80131 Naples, Italy; 2 Dipartimento Medicina Pubblica e della Sicurezza Sociale, Università degli Studi di Napoli ‘Federico II’, Via Sergio Pansini, 5, 80131 Naples, Italy

**Keywords:** Benzene, Pollution, Infertility, IVF, Poor responders, FSH

## Abstract

**Background:**

Several studies have shown that exposure to benzene is associated to menstrual disorders, miscarriages and other disorders of the reproductive system. We performed an observational prospective pilot study to evaluate if levels of benzene in follicular fluid were correlated with response to controlled ovarian stimulation.

**Method:**

Thirty-four normogonadotrophic women undergoing IVF were enrolled. Intra-follicular benzene levels were evaluated by chromatography/mass spectrometry. Based on median benzene level, we divided the study population in two groups: Group A with a “low” intra-follicular benzene concentration (*n* = 19, benzene <0.54 ng/mL) and Group B with a “high” intra-follicular benzene concentration (*n* = 15, benzene ≥ 0.54 ng/mL). The ovarian response to gonadotrophins and the outcome of IVF were analyzed in the two groups.

**Results:**

The two groups did not differ in terms of demographic or anthropometric characteristics. Group B had significantly higher basal FSH levels, lower estradiol peak concentration, and fewer oocytes retrieved and embryos transferred (p < 0.05). Number of gonadotrophin vials, length of controlled ovarian stimulation and ongoing pregnancy rate were similar in the two groups.

**Conclusion:**

In conclusion, ovarian response to endogenous and exogenous gonadotrophins appeared to be influenced by intra-follicular benzene levels.

## Background

Several lines of evidence suggest that exposure to environmental contaminants is involved in the pathobiology of adverse reproductive health effects, including decreased semen quality, sub-fertility, reduced fetal growth and preterm birth [1-6]. Developmental abnormalities of the male reproductive tract were also reported [7]. Among environmental contaminants, polychlorinated biphenyls (PCBs), hexachlorobenzene (HCB), trichloro-bis-chlorophenyl-ethane (DDT) and benzene seem to play a relevant role in adverse reproductive health effects [8].

Benzene is a volatile, colorless, highly flammable liquid [9]. Today, most benzene (98%) is commercially derived from petrochemical and petroleum-refining industries. Benzene is a by-product of such combustion processes as forest fires and the burning of wood, garbage, organic wastes and cigarettes [10-13]. Benzene is ubiquitous in the environment, having been measured in air, water, and human biological samples [13-17].

The major sources of benzene exposure are tobacco smoke, automobile service stations, exhaust from motor vehicles and industrial emissions. About half the exposure to benzene in the United States results from smoking tobacco or from exposure to tobacco smoke [9]. The average smoker (32 cigarettes per day) takes in about 1.8 mg of benzene per day. This amount is about 10 times the average daily intake of benzene by non-smokers [9].

Epidemiologic studies have shown that occupational exposure to high concentrations of benzene negatively affects reproduction. Specifically, benzene exposure in the workplace was associated with high rates of functional disturbances of the menstrual cycle [18], premature interruptions of pregnancy [19,20] and reduction in birth weight and head circumference during pregnancy and at birth [21].

Notwithstanding the evidence linking benzene exposure to negative reproductive health effects, the pathogenesis of adverse reproductive outcomes and benzene exposure has not yet been explored. In this context, *in vitro* fertilization (IVF) is a good *in vivo* model with which to evaluate directly the effects exerted by benzene on each step of the reproductive process.

The aim of this pilot study was to preliminarily evaluate the relationship between concentrations of benzene in the follicular fluid (FF) and the outcome of IVF/ICSI (intracytoplasmic sperm injection) cycles.

## Methods

Thirty-four consecutive women attending the IVF program at the Reproductive Medicine Unit of the University of Naples “Federico II” were prospectively enrolled from November 2011 to January 2012. Institutional Review Board approval was not required for the present study due to the observational design. No adjunctive procedure has been introduced and all the IVF/ICSI cycles were performed according to routine clinical practice.

A comprehensive written consent form was signed by all participants before enrolment. They also completed a short questionnaire regarding their occupation, place of residence, smoking and driving habits. Only normogonadotrophic patients aged < 38 years with menstrual cycles ranging from 24 to 35 days (intra-individual variability ± 3 days), body mass index (BMI) > 20 and < 28.0 kg/m^2^ and hysteroscopic evidence of a normal uterine cavity within the last 6 months were included.

Women with diabetes mellitus, hypopituitarism, hyperprolactinemia, luteal insufficiency, polycystic ovarian syndrome [22], polycystic ovaries, endometriosis, dysthyroidism, chronic inflammatory diseases, autoimmune disorders, chromosomal abnormalities or genetic diseases were excluded.

All patients underwent a gonadotrophin releasing hormone-agonist (GnRH-a) long down-regulation protocol with the administration of GnRH-a triptorelin, at the daily dose of 0.1 mg s.c., from the mid-luteal phase for 12–20 days (Decapeptyl 0.1 mg; Ipsen S.p.A., Milan, Italy). Ovarian stimulation was performed with recombinant follicular stimulating hormone (FSH) (Gonal F, Merck Serono S.p.A., Bari, Italy), using an individualized dose of between 150 IU and 225 IU s.c. daily according to baseline age, BMI and FSH levels. Ovarian response was monitored by ultrasound examination starting on day 5 of stimulation and the dose of recombinant FSH was adjusted if necessary. The ovulatory dose of human chorionic gonadotrophin (HCG), 10,000 IU (Gonasi HP; IBSA Farmaceutici Italia Srl, Lodi, Italy) was administered when at least three follicles reached a mean diameter of 17 mm. Oocytes were retrieved 35 h after HCG prescription.

Oocytes were washed immediately after retrieval in *in vitro* fertilisation medium (IVF Medicult, Copenhagen, Denmark) and cultured before IVF/ICSI. Fertilisation was checked 16–20 h after insemination, which was confirmed by the presence of two pronuclei. Zygotes were then washed and transferred to fresh media for embryo culture. No embryo selection was performed before transfer. Therefore, only embryos obtained after all IVF procedures were implanted. According to Italian legislation our policy was to limit the number of oocytes to be fertilised. Given that young and eumenorrohoic women (good prognosis) were enrolled, 3 oocytes were fertilised in all of them with no case of embryo freezing.

Luteal phase support was given vaginally in the form of 400 mg micronized progesterone (Prometrium; Rottapharm S.p.A., Milan, Italy) twice daily, starting on the day after oocyte retrieval and continuing until the day of the pregnancy test (i.e. day 12 after embryo transfer). The viability of pregnancy was confirmed by transvaginal ultrasound scan at 10 weeks.

### Benzene chromatographic/spectrometric assay

After isolation of oocytes, FF was pooled and stored (−80°C) until analysis. The concentration of benzene in FF samples was measured as follows. Samples were thawed at room temperature and the whole sample (ranging from 500 μl to 2 ml) was transferred into 10 ml vials containing 1 g NaCl. Vials were sealed with silicone/teflon-lined septa, and 50 μl of a 29.6 ng/ml hexadeuterated benzene (C_6_D_6_, internal standard) aqueous solution was added to each sample to obtain a constant internal standard amount of 1.48 ng. Hexadeuterated benzene, sodium chloride, 10 ml vials and silicone/teflon lined (0.1-mm thick coating) septa, “superior standard”, were from Carlo Erba (Milan, Italy). Solid phase microextraction (SPME) fibers, (fused-silica fibers 10 mm long, coated with an 85-μm-thick layer of carboxen/polydimethylsiloxane) and fiber holders were from Supelco (Bellafonte, PA, USA). Samples were analyzed with a head space/solid phase microextraction (HS/SPME) method and gas chromatography coupled with mass spectrometry (GC-MS). We used the Gas Chromatograph-Single Quadrupole Mass Spectrometer “HRGC 8000 series/VOYAGER” (Fisons Instruments, Ipswich, UK), equipped with a split/splitless injector (0.75 mm i.d. inlet liner for SPME [Supelco, Bellafonte, PA, USA]) and a Zebron 624 capillary column (30 m length, 0.25 mm i.d., 1.40 μm thick film [Phenomenex, Torrance, CA, USA]). Briefly, vials were heated at 55°C and kept at this temperature for 30 min, to allow the volatile compounds to reach equilibrium between the gaseous and aqueous phases. Then the SPME device was inserted into the vial and the fiber was exposed to the headspace above the sample for 15 min. Analytes were thermally desorbed by inserting fibers into the gas chromatography (GC) injector at 250°C. The GC oven temperature was kept at 50°C for 2 min, then the temperature was increased to 90°C at 6°C/min and maintained at 90°C for 1 min. Helium (purity: 99.5%) was used as carrier gas, at 1 ml/min constant flow. The mass spectrometry (MS) detector (source temperature, 200°C) was operated in the selected ion monitoring mode. The acquired masses were 51.0, 77.0 and 78.0 m/z for benzene, and 82.2, 84.2 m/z for the internal standard.

Given the small amount of FF samples available, we did not use the standard addition approach generally adopted to quantify benzene in biological matrices. Rather we determined benzene concentration ([B]_s_) by normalizing the benzene chromatographic peak area (Area_BS_) with respect to the internal standard area (Area_IS_) and with respect to the volume (V) of each sample. The following equation was used: [B]_s_ = [(Area_BS_ ⋅ 1.48 ng)/Area_IS_]/V.

### Statistical analysis

The primary study endpoint of this pilot study was the change in the number oocytes retrieved. Secondary endpoints were the mean number of gonadotrophin vials used, the duration of COS, the mean number of embryos transferred and the ongoing pregnancy rate. The population study sample was determined arbitrarily; we were unable to make a pre-study sample size calculation because of the lack of similar studies on this topic.

Results were analyzed using the statistical package SPSS 12.0 for Windows (SPSS Inc., USA). We used the D’Agostino-Pearson normality test to quantify the distribution of all continuous variables. A one-way analysis of variance (ANOVA) was used to determine the effects of stimulation protocols on continuous variables. The Mann–Whitney *U* test was used to assess inter-group differences in relation to non-parametric continuous variables and the *χ*^2^ test to compare discontinuous data. We assessed the relationship between continuous variables and intra-follicular levels of benzene with a linear correlation (Pearson’s correlation), whereas we used Spearman’s coefficient to determine the relationship between intra-follicular levels of benzene and non continuous variables. A *p* value < 0.05 was considered statistically significant.

## Results

The demographic and hormonal characteristics and the indications for IVF/ICSI of the study population are reported in Table 1. Linear regression analysis showed that the intra-follicular level of benzene was significantly and positively correlated with baseline FSH level (*r* = 0.38; *p* = 0.03). Furthermore, it was significantly and negatively correlated with each of the following variables: average number of oocytes retrieved (*r* = −0.43; *p* = 0.010) and average number of embryos transferred (*r* = −0.37; *p* = 0.03). There was no correlation between benzene levels and the following variables: baseline E_2_ (17β-estradiol) levels (*p* = 0.38), baseline LH (luteinizing hormone) levels (*p* = 0.16), E_2_ peak levels (*p* = 0.086), number of exogenous gonadotrophin vials used (*p* = 0.57) and number of days of stimulation (*p* = 0.86).

**Table 1:** The main indications, basal characteristics and the IVF/ICSI outcome of study population

	**Group A (*n*** **= 19)**	**Group B (*n*** **= 15)**	***p value***
*Indications for IVF/ICSI*^*†*^			
**Male factor**	7 (36.8%)	10 (66.6%)	0.16
Severe OAT	6	8	
Mild OAT	1	2	
**Female factor**	10 (52.6%)	5 (33.3%)	0.31
Tubal factor	7	4	
Repeated abortions	1	0	
Others female factors	2	1	
**Idiopathic factor**	2 (10.5%)	0	0.49
*Basal characteristics*			
Age (years)	31 ± 4.7	32 ± 4.8	0.74
BMI (kg/m^2^)	24.0 ± 3.5	25.3 ± 2.2	0.85
Intra-follicular benzene (ng/mL)	0.15 ± 0.2	1.5 ± 1.25	0.001
Baseline FSH (IU/L)	5.8 ± 1.1	11.3 ± 4.2	0.003
Baseline LH (IU/L)	3.9 ± 1.6	8.2 ± 2.5	0.07
*IVF/ICSI outcome*			
Baseline E_2_ (pg/mL)	38.9 ± 16.1	38.4 ± 18.3	0.55
E_2_ peak (pg/mL)	1836.65 ± 661.9	1328.8 ± 493.0	0.008
Days of stimulation	13.2 ± 2.7	13.4 ± 2.6	0.75
Gonadotrophin vials	33.3 ± 15.2	32.65 ± 13.8	0.59
Oocytes retrieved	7.4 ± 2.5	6.6 ± 2.85	0.01
Embryos transferred	2.6 ± 0.4	2.3 ± 0.6	0.04
Ongoing pregnancy rate^†^	4 (21%)	4 (28%)	0.89

Data reported as mean ± SD or percentage [^†^*n* (%)].

BMI, body mass index, FSH, follicle-stimulating hormone; LH, luteinizing hormone, E_2_, 17β estradiol, OAT, oligoasthenoteratozoospermia.

To evaluate the effect of benzene, we divided the study population into two groups using the median benzene concentration (0.54 ng/mL) as cut-off value. Group A (*n* = 19) had levels of intra-follicular benzene < 0.54 ng/mL and Group B (*n* = 15) levels ≥0.54 ng/mL (data not shown). Demographic and anthropometric characteristics did not differ between the two groups (Table 1). No difference regarding main indications to IVF/ICSI was found between groups (data not shown).

There was no statistical difference in terms of baseline LH, baseline E_2_, number of gonadotrophin vials used, and number of days of stimulation between the two groups.

On the contrary, baseline FSH levels were higher in group B than in group A (11.3 ± 4. 2 *versus* 5.8 ± 1.1; *p* = 0.003), and the E_2_ peak was significantly lower in group B (1328.8 ± 493.0 *versus* 1836.65 ± 661.9; *p* = 0.008).

The number of oocytes retrieved (6.61 ± 2.85 *versus* 7.4 ± 2.5; *p* = 0.01) were lower in group B than in group A. Although all women were considered eligible for embryo transfer, the number of embryos transferred was higher in group A, i.e., the group with a low intrafollicular benzene concentration (2.3 ± 0.6 *versus* 2.6 ± 0.4; *p* = 0.04).

Finally, the ongoing pregnancy rate was comparable in the two groups (Table 1). Specifically, we obtained four singleton pregnancies in group A, and three singleton pregnancies and one twin pregnancy in group B.

Based on the replies to the questionnaire, we looked for variables that might account for the differences in the intra-follicular benzene concentration between the two groups. There was no significant difference in terms of place of residence and occupational risk of benzene exposure (data not shown). However, there was a difference, albeit not significant, regarding cigarette smoking (26.3% in group A *versus* 40.0% in group B) and driving habits (68.4% in group A and 46.65% in group B).

## Discussion

This study provides the first evidence that high concentrations of benzene in the ovary can impact negatively on gonadal function and on the ovarian response to endogenous and exogenous gonadotrophin. In particular, a significant correlation was found between intra-follicular levels of benzene and FSH baseline. This correlation was confirmed when the study population was divided into two groups based on intra-follicular levels of benzene; indeed, baseline FSH concentration was significantly higher in patients with higher intra-follicular benzene values (Group B). Also baseline LH level was higher in group B, but the difference was not significant, probably due to the population size.

Consistent with these observations, we also demonstrate that benzene adversely affects ovarian response to exogenous gonadotrophin during IVF procedures. Indeed, there was a statistically significant inverse relationship between the intra-follicular levels of benzene and the average number of oocytes recovered and embryos implanted. This observation was confirmed by the results obtained in the two groups: peak estradiol, average number of oocytes recovered and average number of embryos implanted were significantly higher in group A, which was characterized by low levels of benzene. Nevertheless, the number of gonadotropin vials and the average duration of stimulation did not differ between the two groups. These findings support the hypothesis that benzene could lead to ovarian resistance to exogenous gonadotrophin. Even though a slower and lower response to r-hFSH occurred in the high benzene group, oocyte retrieval was apparently satisfactory and the daily dose of exogenous gonadotrophin was not increased in the “high” benzene group. A possible explanation of this discrepancy is that benzene exerted local toxicity on follicles thereby affecting the number observed during scan monitoring and, to a greater extent, their subsequent maturation process. This discrepant effect could also be related to the characteristics of the study population and of course to the small number of patients enrolled in the study. According to our findings, it seems that the normal ovarian reserve probably counteracted the detrimental effects of benzene, leading to a sort of “hypo-response” rather than the classical “poor ovarian response” [23]. However, given the low number of patients evaluated, this hypothesis needs to be verified in larger trials.

Our data are in accordance with observational studies conducted in a business environment which revealed that exposure to benzene can negatively affect reproduction function [24-26].

However, using the model of IVF/ICSI we show, for the first time, that high intra-ovarian levels of benzene are associated with hypo-sensitivity of follicles to gonadotrophin stimulation. The mechanism underlying this phenomenon is not clear. We may speculate that ovarian resistance results from an alteration of transductional efficiency of FSH receptor or that benzene exerts a degenerative effect on follicles, thereby reducing the follicular reserve.

If our findings are confirmed, the next step would be to evaluate the ovarian reserve in patients with high intra-ovarian levels of benzene by measuring circulating levels of anti-Müllerian hormone and by counting the number antral follicles in early follicular phase using trans-vaginal standard and three dimension ultrasound [27,28]. In addition, it would be interesting to evaluate older patients to determine whether age affects benzene toxicity. Unfortunately, we were unable to perform this analysis because of the small subgroup of cases above the age of 35 years old.

Although the present study provides information only about oocyte quantity during IVF, it could be argued that altered sensitivity to FSH is associated with reduced oocyte quality, which in turn could affect the chances of pregnancy and the risk of abortion [29]. This issue should be addressed in a prospective trial performed in a larger IVF study population, with the pregnancy rate as primary endpoint.

The subjects enrolled in this study completed a questionnaire designed to explore the link between life-style and concentration of benzene in the ovary. Currently, there is no standardized questionnaire that reliably assesses benzene assumption and driving habits. The reliability of self-reports of smoking is often questioned due to the underestimation of the smoking status recorded especially in communities where this habit is perceived as being particularly improper [30]. Despite this relevant bias and a sample size too low to detect a statistically significant correlation, our data suggest that high intra-follicular levels of benzene are related to an at-risk life-style, namely, cigarette smoking.

The aim of our study was to investigate the direct effect of follicular benzene on the outcome of COS. We did not evaluate serum levels of the hydrocarbon molecule at that stage. Should our data be confirmed, the next step would be to investigate the relationship between serum and follicular concentration of benzene. This information could shed light on the potential role of serum levels of benzene in predicting ovarian response.

## Conclusion

Overall, the results of this study, although preliminary, support the existence of a link between exposure to benzene and reduced reproductive capacity, and provide evidence of follicular hyposensitivity to FSH in women with high gonadal concentrations of the molecule. The next step would be to define the pathogenetic mechanisms underlying resistance to endogenous gonadotrophin and the reduced ovarian response to exogenous gonadotrophin. Comparative analysis carried out in large populations using the *in vitro* fertilization model would enable an accurate assessment of the effects of benzene on each stage of the reproductive process.

## Abbreviations

BMI: Body mass index; COS: Controlled ovarian stimulation; DDT: Trichloro-bis-chlorophenyl-ethane; E_2_: 17β-estradiol; FF: Follicular fluid; FSH: Follicle stimulating hormone; GC: Gas chromatography; GC-MS: Gas chromatography coupled with mass spectrometry; GnRH-a: Gonadotrophin releasing hormone-agonist; HCB: Hexachlorobenzene; HCG: Human chorionic gonadotrophin; HS/SPME: Head space/solid phase microextraction; ICSI: Intracytoplasmic sperm injection; IVF: *In vitro* fertilization; LH: Luteinizing hormone; MS: Mass spectrometry; OpU: *Ovum* pick-up; PCB: Polychlorinated biphenyls; SPME: Solid phase microextraction; V: Volume; 3D: Three dimension.

## Competing interests

The authors declare that they have no competing interests.

## Authors’ contributions

CA was the principal investigator for this study and wrote the first manuscript draft. RG and AA carried out experiments on follicular fluid for benzene quantification. GC, PD, RV and TP were involved in patient recruitment and collection of data. AC, SP, GC, IS and AM performed COS and actively contributed to interpretation of findings. GD and AA supervised the manuscript. All authors actively contributed to the manuscript and gave final approval of submitted version.
